# Promotion of exclusive breastfeeding among HIV-positive mothers: an exploratory qualitative study

**DOI:** 10.1186/s13006-016-0068-7

**Published:** 2016-04-20

**Authors:** Alice N. Hazemba, Busisiwe P. Ncama, Sello L. Sithole

**Affiliations:** Department of Public Health, School of Medicine, University of Zambia, Lusaka, Zambia; School of Nursing and Public Health, Howard College Campus, University of KwaZulu Natal, Durban, South Africa; Department of Social Work, School of Social Sciences, University of Limpopo, Sovenga, South Africa

**Keywords:** Exclusive breastfeeding, Formula feeding, Promotion, HIV-positive, Informed-decision, Zambia

## Abstract

**Background:**

Exclusive breastfeeding has the potential to reduce infant and under-five mortality, but research shows the practice is not widespread in resource-poor settings of sub-Saharan Africa. We explored factors influencing the decision to exclusively breastfeed among HIV-positive mothers accessing interventions for prevention of mother-to-child transmission of HIV in selected sites of Zambia.

**Methods:**

This exploratory qualitative study was embedded in research conducted on: HIV and infant feeding; choices and decision-outcomes in the context of prevention of mother-to-child transmission among HIV-positive mothers in Zambia. Thirty HIV-positive mothers and six key informants were recruited from two health facilities providing mother-to-child HIV transmission prevention services. A semi-structured guide was used to conduct interviews, which were digitally recorded and simultaneously transcribed. Data coding and analysis was done with the support of QRS Nvivo 10 version software.

**Results:**

Despite the known benefits of exclusive breastfeeding, gaps in understanding and potential for behaviour change remained. We found that information promoting exclusive breastfeeding may have been understood by mothers as instructions from the health care workers indicating how to feed their HIV-exposed babies rather than as an option for the mothers’ own informed-decision. This understanding influenced a mother’s perceptions of breast milk safety while on antiretroviral medicine, of the formula feeding option, and of the baby crying after breastfeeding. The meanings mothers attached to exclusive breastfeeding thus influenced their understanding of breast milk insufficiency, abrupt weaning and mixed feeding in the context of preventing mother-to-child transmission of HIV.

**Conclusion:**

In order to enhance feeding practices for HIV-exposed infants, our study suggests a broader health campaign supporting all mothers to exclusively breastfeed.

## Background

Breastfeeding is a way of providing ideal food for the healthy growth and development of infants and in some settings of the sub-Saharan Africa it is a customary way of feeding new-born babies [1, 2]. However, mothers known to be infected with HIV risk transmitting the virus to their babies through breast milk, which has changed the landscape of infant feeding in sub-Saharan Africa [3]. Research has shown that breastfeeding – especially exclusive breastfeeding, limited to breast milk only, plus any minerals, vitamins and prescribed medicines that may be needed – for the first six months after birth reduces the risk of postpartum transmission of HIV from an infected mother to her baby [4–7]. Non-exclusive breastfeeding, on the other hand, more than doubles the risk of vertical transmission of HIV [8–10]. Despite the recognized importance of exclusive breastfeeding, the practice is not widespread in the developing world [11–13]. To practice exclusive breastfeeding, HIV-positive mothers may have to go against cultural norms that support early introduction of fluids and mixed feeding [1, 14, 15].

In Zambia, the prevalence of HIV among women aged between 15 and 49 is currently 15 per cent [16]. According to the Ministry of Health (MoH) report, a decline was recorded in the number of new HIV infections among children younger than 15 years from 19,000 in 2009 to 9500 in 2012 [17]. Reductions have also been recorded in the HIV transmission rate from mother-to-child, during breastfeeding, from 24 per cent in 2009 to 12 per cent in 2012, representing a 51 per cent decrease in the number of new HIV infections among children [17]. The MoH further states, however, that 5 in 10 HIV-positive women or their infants did not receive antiretroviral drugs during breastfeeding to prevent mother-to-child transmission of HIV [17].

Without stepping up interventions, babies born to HIV-positive women will continue to have added risk of acquiring HIV infection and dying from it before their fifth birthdays [18]. As a public health approach, all mothers in Zambia are encouraged to practice exclusive breastfeeding for 6 months regardless of their HIV status [17, 19]. Additional interventions addressed in the roll-out of the 2013 Zambian consolidated guidelines for mothers infected with HIV were prevention of mother-to-child transmission (PMTCT) and provision of life-long combination antiretroviral therapy (ART) regardless of CD4 count (Option B^+^) for pregnant and breastfeeding mothers [20].

Coupled with promotion of exclusive breastfeeding and life-long ART for HIV-positive mothers is the need for objective counselling which assesses the suitability of the different feeding options for each mother and gives her the information she needs to make a genuinely informed-decision about how she will feed her HIV-exposed baby [21] – a point which is emphasised in the World Health Organization (WHO) infant feeding guidelines [2]. The aim of this sub-study was to explore factors that influence the decision to exclusively breastfeed in the context of preventing mother-to-child transmission of HIV.

## Methods

### Design

This exploratory qualitative study formed part of a research project titled “HIV and infant feeding; choices and decision-outcomes in the context of prevention of mother-to-child transmission (PMTCT) among HIV-positive mothers in Zambia”. The study aimed to analyse infant feeding choices and decision-outcomes in the context of PMTCT and enhance safer infant feeding practices during the first six months of the infant’s life. Data collection occured through individual interviews with mothers and key informants. Attendance at individual infant feeding counselling sessions was not part of the scope of this study. After giving birth, mothers were interviewed at six days, six weeks, twelve weeks and eighteen weeks. This paper addresses the theme: promotion of exclusive breastfeeding among HIV-positive mothers accessing PMTCT services.

### Setting

The study was conducted in an urban setting of Lusaka district using two health facilities to recruit study participants. The health facilities are sites for Ministry of Health (MoH) PMTCT and ART programmes integrated in maternal, neonatal and child health (MNCH) services. The fieldwork was conducted from January to September 2014.

### Participants and sampling

The main study focussed on a group of 30 HIV-positive mothers, selected by purposive sampling, who were accessing PMTCT services. In addition, six key informants were selected from the PMTCT programme.

For the group of HIV-positive mothers, selection criteria were: a mother should have attended health promotion talks on infant feeding, been tested for HIV, been placed on treatment regimen (Antiretroviral medicines), been counselled on infant feeding, opted to exclusively breastfeed, and have a normal vaginal delivery of a live full-term baby. Categories such as education level and socio-economic status were considered during sampling as themes emerged because the study sites catered to women of differing socio-economic background.

### Data collection

A team, composed of the principal investigator, who is the first author, and two research assistants (RAs) who were qualified and practicing midwives conducted data collection. The RAs were conversant in the primary language spoken by mothers, trained in counselling and PMTCT interventions. The first author planned and conducted training of the RAs regarding the use of a data collection guide, procedures for recruitment of participants, management of consent and issues of confidentiality, interviewing techniques, and management of audio recordings.

A semi-structured interview schedule was used to collect data. The main interview question for health care workers (HCWs) was: “how have you been presenting the messages on promotion of exclusive breastfeeding?” The main interview questions for HIV-positive mothers were: “tell me what you know about exclusive breastfeeding” and “tell me why you chose to exclusively breastfeed your baby, and how have you fed your baby since giving birth?”

### Key informant interviews

Key informant interviews were conducted with two nurses, two nutritionists and two clinical officers. These interviews were conducted one time at the beginning of data collection to develop the questions to be asked of the HIV-positive mothers. These HCWs provided the information, ideas and insights in the promotion of exclusive breastfeeding in relation to PMTCT.

### Participant observation

Participant observations were undertaken as a complementary method and were essential in detecting meanings and feelings attached to breastfeeding [22]. Situations that provided an opportunity for participant observations included health education sessions where HCWs informed mothers about exclusive breastfeeding and its benefits to infants regardless of the HIV status of the mother. These health education sessions were part of routine activities in the MNCH units at the health facilities. The observations were extended to homes with permission from mothers who preferred to be interviewed from their home environments.

### Individual interviews

Individual interviews were considered an appropriate method because they made it possible to effectively address complex and sensitive topics and allowed mothers to talk about personal feelings, opinions and experiences using their primary language.

### Data management

All audio recordings were transcribed verbatim from primary language into English. All transcripts were checked for accuracy and quality. All identifiers were removed to insure anonymity and all the files were imported into QRS Nvivo 10 version for coding and analysis.

### Ethics

Ethical approval was granted by the Humanities and Social Sciences Research Ethics Committee of the University of KwaZulu-Natal in South Africa (HSS/0104/013D) and the Biomedical Research Ethics Committee of the University of Zambia (Reference No. 016-11-13). All participating women and key informants gave written and signed consent.

### Data analysis

Using framework analysis, data analysis proceeded alongside data collection [23, 24] and emerging themes were followed up in subsequent interviews. Interpretation of the results was guided by social constructivism, a theory of knowledge construction articulated by Schwandt [25] in which the basic tenets are that knowledge is formed by people in their daily interaction with one another and that knowledge is a social product. Mothers in this study constructed their own knowledge, based on their understanding of information on exclusive breastfeeding presented by HCWs. We thus relied as much as possible on the mothers’ views and understanding of exclusive breastfeeding formed through interactions with HCWs, although our interpretations of the mothers’ understanding and behaviour in relation to PMTCT of HIV were also shaped by our own background and experience with HIV/AIDS interventions. We formed a consensus construction that was largely based on the perspectives of mothers and of the researchers [25].

At inception of data collection, the analytical memos in the initial coding phase were used to identify codes relevant to exclusive breastfeeding in the context of PMTCT. The meanings that mothers attached to exclusive breastfeeding were diverse. This led us to look for the complexity of views rather than narrow the meanings into a few categories or ideas. The integrated data on major themes were further analysed for varying and similar perspectives in order to develop sub-themes as appropriate. In all the themes, quotes (constructs) of the mothers’ actual words were linked to the descriptions [26].

## Results

The analysis identified four major themes that related to factors influencing the decision to exclusively breastfeed in the context of PMTCT as: (i) promotion of exclusive breastfeeding by HCWs; (ii) the mothers’ understanding of information to exclusively breastfeed; (iii) the mothers’ reasons for choosing to exclusively breastfeed; and (iv) decision making on infant feeding and behaviour change in relation to PMTCT. These themes are described in detail in the next section of the article.

### Demographic characteristics of mothers interviewed

The mothers accessing PMTCT services and who were our target group from the selected sites, self-reported as married; two reported as single and one reported as a widow. Nearly all had lower primary education and were unemployed (Table 1).

**Table 1:** Demographic characteristics of mothers interviewed

Characteristic	Categories	Frequency
Age	15–24	10
	25–34	16
	≥35	4
Marital status	Single	2
	Married	27
	Widowed	1
Education (Grades)	0–7	14
	8 & 9	12
	10–12	2
	College education	2
Employment	None	28
	Employed	2
Number of children	1–3	21
	4–6	9

### Key informants interviewed

Six HCWs were recruited for the study. The nurses (*n* = 2) were key providers of MNCH services, the nutritionists (*n* = 2) were involved in counselling for infant and young child feeding, and the clinical officers (*n* = 2) provided screening, diagnosis and treatment services. All the HCWs in the selected sites were orientated to PMTCT interventions and trained in HIV counselling and testing. The key informant interviews were conducted at the inception of the fieldwork to clarify the questions to be used during interviews with HIV-positive mothers. When it became apparent that emerging themes from individual interviews with mothers needed clarification, these key informants were interviewed to shed more light on the subject under investigation. This was done until the interviews reached saturation stage where new information could not be generated.

### Promotion of exclusive breastfeeding by Health Care Workers

According to the 2010 Ministry of Health national infant feeding guidelines for Zambia, all mothers are encouraged to exclusively breastfeed their babies for the first six months of life [19]. In this study, we observed that group health education was conducted during antenatal care to encourage all attending pregnant women to practice exclusive breastfeeding regardless of their serostatus. Group health education was the first activity in the counselling process for HIV-positive mothers. This activity was conducted before mothers could be provided with other pertinent and more specific services such as HIV counselling and testing, infant feeding counselling as well as other antenatal care (ANC) services. Therefore, all mothers attending ANC for the first time (antenatal booking) and through subsequent visits had an opportunity to listen to these counselling talks. HIV-positive mothers were additionally given individual training on infant feeding.

Although information was given to HIV-positive mothers during individual counselling sessions about the other available infant feeding option (formula feeding) for HIV-exposed infants, the HCWs interviewed reported encouraging mothers to practice exclusive breastfeeding as a policy directive from the Ministry of Health. This led us to conclude that mothers were not given an opportunity to weigh the pros and cons of other feeding options thus denying them the opportunity to make an informed-decision in total disregard of their socio-economic situation which would not have allowed them to initiate and sustain formula feeding. The HCWs understood the promotion of exclusive breastfeeding in this context as a matter of prescribing in an effectively top-down approach. In the words of a key informant with two years of experience in PMTCT: “*We tell HIV-positive mothers from the scientific point of view on what is expected. We encourage them to follow national guidelines on infant feeding regardless of what their families or people in the community expect of them. We emphasise what is supposed to be followed and if they exactly follow this (instructions), there won’t be any problems”.*

Despite the WHO recommendation/s and Ministry of Health guidelines to assess acceptability, feasibility, affordability, sustainability and safety (AFASS) when discussing formula feeding, the responses by key informants were confined only to determining whether the mother could afford the formula. Asked whether mothers were informed about formula feeding or other available infant feeding options, a key informant explained, for instance, that although formula feeding was discussed, the emphasis was to assist mothers to exclusively breastfeed: *“We explain to mothers that because of their HIV-positive status, they have two choices on how to feed their babies. That is, either to breastfeed exclusively or to give formula. If they can manage and afford to buy formula for the first six months of the baby’s life then they can choose formula. But we emphasise that if they exclusively breastfeed and adhere to medication (ARVs), the risk of the baby getting infected with HIV through breast milk are very, very slim”.*

Although the PMTCT intervention allows mothers to make an informed-decision on infant feeding and the process is clearly defined in the national and WHO guidelines, mothers in this study reported that they were not assisted step by step in making their decision on how to feed their babies. Consequently, mothers were reported as doubting the risk of mother-to-child transmission (MTCT) would be reduced while they were on ARVs and exclusively breastfeeding. This was highlighted in another key informant interview:

*“HIV-positive mothers make an informed-decision on infant feeding although sometimes they don’t believe that if they continue breastfeeding exclusively while they are taking their medication (ARVs) the possibility of them passing the HIV virus to the baby through breast milk is reduced. They think that the baby can still easily get infected”.*

These disclosures led us to conclude that HCWs seeking to discourage formula feeding, needed nonetheless to give a clear explanation of exclusive breastfeeding practice in relation to the AFASS criteria of acceptability, feasibility, acceptability, sustainability and safety. Although HCWs recognised the importance of informing mothers about the advantages and disadvantages of exclusive breastfeeding and formula feeding, their perspectives leaned more towards promotion of exclusive breastfeeding only, and comments were made such as ‘*we are not giving them options*’, ‘*options are not discussed*’, and ‘*at the moment we are left with exclusive breastfeeding*’. Such comments were highlighted in the following responses:

*“We are not giving options because mothers are following the national guidelines. All babies despite their mothers’ HIV status are to be exclusively breastfed from zero up to six months. We also offer support to mothers to exclusively breastfeed”.* [nutritionist, 5 months in PMTCT]

*“The options on infant feeding are not discussed because we fear that we may discourage a mother that would really want to breastfeed. This is how we talk to them even on a one-to-one basis. During one-to-one counselling sessions, we give mothers information about the benefits and disadvantages of breastfeeding to the baby and the mother. We emphasise that Zambia is promoting exclusive breastfeeding regardless of the HIV status of the mother, but the mother will finally make a decision on how to feed her own baby”.* [nutritionist, 4 years in PMTCT]

*“We are talking to all mothers during group health education (group counseling) and individual counselling sessions on infant feeding but as far as choices are concerned, at the moment we encourage exclusive breastfeeding for the first 6 months regardless of the HIV status of the mother. This is the way we’ve gone”.* [nurse, 15 years in PMTCT]

To adequately provide individual infant feeding counselling, the mothers need appropriate health facilities that provide adequate space and trained HCWs. A nutritionist key informant who had worked for four years providing counselling on infant and young child feeding commented that a conducive environment was needed for promoting exclusive breastfeeding, such as scheduling counselling sessions at the health facility at times that would encourage seropositive mothers to meet, and see for themselves, other mothers breastfeeding their babies who are in the same situation:

*“The health facility itself needs to have a schedule in place for teaching HIV-positive mothers to promote exclusive breastfeeding and to support mothers during infant feeding. Mothers need to be motivated to protect their decisions to exclusively breastfeed and they should find a friendly environment where they will see their fellow women breastfeeding as well. This will serve as a motivation despite their serostatus. They’ll also learn from their friends’ experiences on breastfeeding”.*

Key informants reported that it was also useful to have health education materials such as brochures in primary languages spoken by mothers available as carry-home materials to read and to conduct demonstrations on breastfeeding:

*“When we have them, we give mothers brochures so that they can read in their primary languages. We show mothers some materials with the actual pictures of mothers feeding their babies. We also conduct demonstrations on breastfeeding right here at the health facility”.*

### The mothers’ understanding of information to exclusively breastfeed

The aim of promotion of exclusive breastfeeding is to improve child survival for the HIV-exposed babies. In this study, mothers appeared to understand that the ARVs they were taking reduced the risk of MTCT of HIV through breast milk and hence recognized its benefits for their exposed babies. This understanding was in line with what was reported by key informants. Typical responses by mothers were:

*“The medicine [ARVs] that I am taking is helping me to exclusively breastfeed and the baby also needs to take the medicine (ARVs) which we get from the clinic”.* [mother, 23 years, 1 child]

*“The risk of infecting the baby with HIV is reduced if the baby is breastfed only and as long as I am taking the medicine [ARVs] as instructed by the nurses”.* [mother, 34 years, 4 children]

Although the HCWs declared that promotion of exclusive breastfeeding was meant to assist mothers to make an informed-decision on how best to feed their babies, it seems likely that mothers took the information they received in the counselling sessions as directive rather than informative from which they were free to make their own decisions. Promotion of exclusive breastfeeding in the context of informed-decision implies that the choice is made by a counselled (informed) HIV-positive mother, assisted by relevant and context-appropriate health-promotion messages, with full knowledge about the advantages and disadvantages of the possible feeding options being promoted. However, the word ‘just’ was used frequently by mothers when referring to the decision to exclusively breastfeed as shown in the following responses:

*“I just had to exclusively breastfeed because the nurses are the ones to tell me everything because I am not educated”.* [mother, 25 years, 1 child]

*“I just decided based on what they taught us at the clinic. To give the baby breast milk only for six months”.* [mother, 33 years, 4 children]

*“I don’t have much information, because I just know that I have to breastfeed the baby without giving him anything else as advised by the nurses, things like that”.* [mother, 22 years, 1 child]

*“At the clinic, they just said that the baby should be breastfed for six months without giving him any other foods, only breast milk”.* [mother, 32 years, 2 children]

Frequent use of the word ‘just’ by mothers signifies the absence of choice in feeding their babies. It drives home the fact that the approach adopted and provided by HCWs was more prescriptive rather than encouraging choice of options.

### The mothers’ reasons for choosing to exclusively breastfeed

Besides the information on promotion of exclusive breastfeeding for HIV-exposed infants, there appeared to be other factors influencing mothers’ decision to exclusively breastfeed. Factors that came into consideration for the mothers were understanding of breast milk safety versus formula for their HIV-exposed babies, perception of baby’s crying after breastfeeding, and their ability to meet and sustain the cost of formula. The following subthemes describe each of the highlighted factors with corresponding responses.

### The mothers’ understanding of the safety of breast milk versus formula

The HCWs in this study reported emphasising to mothers that exclusive breastfeeding while adhering to ARV protocol reduced the risk of MTCT through breast milk. Despite this emphasis, some mothers doubted the safety of breast milk for their HIV-exposed babies even when taking ARVs, while others regarded it as a safer option given their individual circumstances. These two divergent perspectives on the part of mothers may have arisen from inadequate information and lack of understanding of exclusive breastfeeding practice in the context of PMTCT of HIV.

These perspectives were apparent when mothers were asked about what their decision to exclusively breastfeed meant to them and their babies. A 35 year-old mother of four children feared that through breast milk, she may infect her baby with HIV: “… *just not wanting to breastfeed due to fear that the baby may be infected with HIV through breast milk”*. Other mothers, who could afford formula, were aware of the risks of waterborne diseases suggesting that women are considering risks associated with formula when making their decisions. A mother explained: “*I made a choice to breastfeed my baby because my other babies who I gave formula passed on. The formula used to give them diarrhoea till they died, and for this reason, I decided to breastfeed this baby exclusively without giving him any other fluids or food”* [mother, 32 years, 1 child]*.* Even when the cost of formula was not a deterrent, a 32-year-old mother of 4 children, employed as a teacher, weighed formula feeding against the protective effect of breast milk for her new-born baby: *“Of course I can manage to buy the formula. It’s not because of money, I chose to exclusively breastfeed in order to protect my baby from other diseases”.*

### The mothers’ ability to meet and sustain the cost of formula

Whereas breastfeeding was thought to be cheap and always available on demand, mothers were also aware of the risks of mixed feeding of formula and breast milk. A 26-year-old mother of two children explained: “*I chose to breastfeed because it is cheap and I was not scared after being given the information that the baby cannot get sick if it is exclusively breastfed. I was more scared to kill my baby with hunger because I may not have enough money to buy formula”.* She added: *“I can’t also be changing from giving my baby formula today, and then tomorrow if the formula finishes I give the baby breast milk. That is why I chose to exclusively breastfeed”.*

The following responses confirmed that the cost of formula was a deterrent to choosing exclusive breastfeeding:

*“I decided to breastfeed the baby because we [with spouse] did not have enough money to buy formula, and there was nothing else I could do”.* [mother, 31 years, 5 children]

*“If I could manage to buy formula, I would have chosen to give formula and not breastfeed”.* [mother, 26 years, 4 children]

*“If I had money, I would buy formula and not breastfeed”.* [mother, 35 years, 4 children]

### Decision making on infant feeding and behavior change in relation to PMTCT

According to the HCWs, promotion of exclusive breastfeeding is not confined to teaching and needs to include support for mothers during the infant feeding period, especially the first six months after giving birth. When we interviewed mothers to explore how messages promoting exclusive breastfeeding contributed to their understanding and behaviour in relation to PMTCT during the first six months of infant feeding, additional hindrances to exclusive breastfeeding practice emerged. These included perceived breast milk insufficiency when the baby cried after breastfeeding, mixed feeding and potential for abrupt weaning due to breast complications.

### Perceived breast milk insufficiency

Despite the promotion of exclusive breastfeeding for HIV-exposed infants, there emerged other factors that influenced the way mothers regarded exclusive breastfeeding practice. The perception by some mothers that babies cry of hunger because the breastmilk is sometimes not enough influenced how they interpreted the baby crying even after breastfeeding. This perception, along with limited understanding of how to manage infants with childhood ailments, could potentially induce mothers to mix the feed with light porridge, as explained by a 25-year-old mother of one child: *“Sometimes you know how the devil is, the baby is crying and maybe it is hungry, you sense the temptation to make some light porridge and give the baby so that it can stop crying. But at the same time you know the instructions from the clinic, where they said the baby should only be given food and water after six months”.* Another mother added: *“When the baby cries a lot, it can force you to give it anything. You can make the baby drink anything available so that it stops crying, because sometimes you don’t know why it is crying… maybe it is hunger. You can be forced to do something for the baby”.* [mother, 21 years, 2 children]

Asked how they were feeding their babies, some mothers reported giving their babies other fluids apart from breast milk. A young first-time mother explained: “*Yes I am breastfeeding her but if I have gone very far, she takes Bonita milk (Cow’s milk). I give her from the cup. The breast milk is not enough that is why I mix with Bonita”.* [24 years, 1 child]. Another mother added: “*I give orange juice to the baby and something for the stomach apart from breastfeeding”.* [mother 22 years, 2 children]

### Potential for abrupt weaning from exclusive breastfeeding due to breast complications

It also emerged from the interviews with mothers, that they were unclear about weaning practices and under what circumstances weaning might be appropriate. In the event that sores developed on the breasts of the mother, this potentially altered their capacity to exclusively breastfeed and as a consequence, abrupt weaning from exclusive breastfeeding was planned or initiated. Typical responses were:

*“Sometimes I think that I may have a sore on my nipples which I may not be aware of. But the nurses said that there should not be any sores on the breast when breastfeeding. So I get worried that the baby may get infected with HIV”.* [mother, 26 years, 2 children]

*“I usually think about weaning my baby from breastfeeding, since the nurses said that at times there can be sores on the mother’s nipples”.* [mother, 32 years, 4 children]

*“The nurses said that I should breastfeed the baby and if sores develop on my breasts, I should not breastfeed him. They said I need to go back to the clinic to inform them”.* [mother, 26 years, 4 children]

The lack of understanding of weaning practice was further highlighted by a young mother who explained that being bitten on the nipples by the baby would warrant giving formula until the sores healed. She explained: “*When the baby starts teething and during breastfeeding it bites you on the nipples then you need to stop giving the baby breast milk and give formula until that sore heals”.* [mother, 23years, 2 children]

## Discussion

The study revealed that despite information promoting exclusive breastfeeding for HIV-exposed infants, problems remained among HIV-positive mothers in their understanding of the information impacting their willingness to change behavior. Exclusive breastfeeding for the first six months is known to reduce the risk of postnatal vertical transmission of HIV (infection) from mother-to-child [4, 5, 7]. Although mothers reported that they were aware that exclusive breastfeeding while on ARVs reduced the risk of MTCT of HIV through breast milk, we found that the messages promoting exclusive breastfeeding may have been understood simply as instructions from the HCWs indicating how to feed HIV-exposed infants rather than knowledge imparted to enable an informed-decision. This was evident when mothers doubted the safety of breast milk while on ARVs and their limited understanding of the formula feeding option.

According to the WHO infant feeding guidelines, mothers known to be HIV-infected should be informed of the infant feeding practice recommended by the national authority, both in order to improve HIV-free survival of exposed infants and so that the mothers are aware of alternatives they might wish to adopt [2, 19, 20]. Although clearly defined, counselling on methods of infant feeding in PMTCT programmes have shown practical and theoretical variations by sites [27–29]. In this study, both HCWs and mothers used expressions that implied direct instruction to exclusively breastfeed rather than imparting knowledge about exclusive breastfeeding as recommended in the current national infant feeding guidelines. This indicates that HCWs need to be fully orientated to PMTCT interventions, be equipped with appropriate communication skills, spend time with mothers focusing on individual needs, and help them to make a genuinely informed-decision to practice exclusive breastfeeding.

Promotion of exclusive breastfeeding is aimed at child survival in Zambia, where infant and under-five mortality is high, 45 and 75 per 1000 live births respectively [16]. In developed countries, mothers that are infected with HIV will more likely choose formula. But for their poorer counterparts, such as the women who were interviewed for this study and others in similar settings, exclusive breastfeeding becomes appropriate in view of poor sanitary conditions and the probability of inappropriate use of formula that can lead to diarrhoea and dehydration; the major causes of infant mortality globally [3, 18]. It is in this context, therefore, that health promotion messages for HIV-positive mothers to practice exclusive breastfeeding need to be understood. Studies in various settings of sub-Saharan Africa have shown that more is needed than just one session promoting exclusive breastfeeding if the recommendations are to achieve their intended goal [30, 31]. Addressing national authorities, Lazarus, Struthers and Violari [32] argued strongly that a top-down instructive approach is not enough to bring about necessary behaviour change in the way HIV-exposed infants are fed in resource-poor settings. They recommended an open and sustained engagement facilitating dialogue about infant feeding with HIV-positive mothers, their families, communities and even HCWs. Despite the known benefits of exclusive breastfeeding while the mother is on ARVs, mothers in this study did not have a sustained engagement with HCWs on infant feeding counselling in order to fully understand and practice exclusive breastfeeding because they only had two sessions. As observed in similar settings, it is crucial for infant feeding counselling messages to be specifically aligned not only to local context [33], but also with an environment that enables mothers to understand the recommended infant feeding options for HIV-exposed infants so that informed-decision can be made [34–36].

Challenges thus remain on how to ensure that HIV-positive mothers understand exclusive breastfeeding practice in the context of PMTCT and are adequately prepared to manage problems that may arise during infant feeding period. Considering that almost all participating mothers had primary and lower secondary education and in view of the country’s high illiteracy levels [16], more women accessing PMTCT services in Zambia will need a schedule of activities that will address challenges faced during exclusive breastfeeding. However, like other countries in sub-Saharan Africa, poor staffing levels, lack of infrastructure and inaccessible health promotion materials pose challenges to adequately provide infant feeding counselling and support; especially where exclusive breastfeeding is concerned [37–39].

Knowledge gaps due to inadequate infant feeding counselling may have contributed to the mothers’ perceived breast milk insufficiency associated with the baby crying even after breastfeeding. This finding is consistent with research in similar settings such as Uganda [40], Malawi [41], Swaziland [42], and was found to be a persistent feature in Zambia [43]. The perception that some breasts produce more milk than others could thus, potentially, be thought to justify mixed feeding of breast milk with other fluids and foods. Although we did not find any strong research to support the theory about some breasts producing more milk than others in similar settings, some scholars suggest that mothers should be taught how to effectively position and attach their infants to the breast to optimise breastfeeding and counter perceptions of milk insufficiency. This is on the basis that unrestricted exclusive breastfeeding results in ample milk production [44]. Although, HCWs reported that they supported mothers to choose and practice exclusive breastfeeding, mothers in this study lacked the necessary skills needed to successfully feed their infants as recommended. As reported in other settings, this could have been due to low staffing levels, inadequate infrastructure, lack of communication skills among HCWs, slow updates on infant feeding guidelines and to a certain extent poor staff attitudes [29, 40, 45, 46]. To address some of these challenges, HCWs in the current study reported that they used demonstrations to teach mothers how to put the baby to the breast but may not have had time to supervise each of them and make sure that they had acquired the skills which they needed to apply in their home settings. The low staffing levels in health facilities in Zambia could thus impede these efforts [47].

We recognise that even though it is a natural act, breastfeeding has also been described as a learned behaviour [48] and that mothers need to be equipped with information and skills to feed their infants and wean them appropriately. Regarding cessation of breastfeeding, the WHO infant feeding guidelines advise that mothers known to be HIV-infected should stop gradually over the course of a month, while continuing to receive support and treatment, and that mixed feeding should be discouraged [2]. This research showed that some mothers are not understanding the risks of mixed feeding and in this society, it is considered a cultural norm [43] and has been reported in similar settings of sub-Saharan Africa [14, 15, 49–51]. Provided they have accurate information, mothers in this and similar settings need to be supported by the health care system and by family and community in initiating and practicing exclusive breastfeeding [48, 52, 53] for their HIV-exposed infants.

## Conclusion

Although it does not attempt to generalize the findings to a larger population, our study suggests a broader health promotion campaign supporting all mothers to exclusively breastfeed. More effort is needed to improve communication skills among HCWs to enable them provide objective infant feeding counselling regardless of the method of feeding promoted for HIV-positive mothers.

## Abbreviations

AFASS: acceptable, feasible, affordable, sustainable, safe
AIDS: acquired immune deficiency syndrome
ANC: antenatal care
ART: antiretroviral therapy
ARVs: antiretroviral
HCWs: Health Care Workers
HIV: human immune virus
MNCH: maternal neonatal and child health
MoH: Ministry of Health
MTCT: mother-to-child transmission
PMTCT: prevention of mother-to-child transmission
RAs: research assistants
WHO: World Health Organization
